# Long COVID and its Management

**DOI:** 10.7150/ijbs.75056

**Published:** 2022-07-11

**Authors:** Ho Cheng Koc, Jing Xiao, Weiwei Liu, Yong Li, Guokai Chen

**Affiliations:** 1 Centre of Reproduction, Development & Aging, Faculty of Health Sciences, University of Macau, Taipa, Macau, China; 2 Institute of Translational Medicine, Faculty of Health Sciences, University of Macau, Taipa, Macau, China; 3 Interventional Medical Centre, Zhuhai People's Hospital, Zhuhai Hospital Affiliated with Jinan University, Zhuhai 519000, China; 4 Bioimaging and Stem Cell Core Facility, Faculty of Health Sciences, University of Macau, Taipa, Macau, China

**Keywords:** COVID-19, SARS-CoV-2, SARS, long COVID, post-acute sequelae of SARS-CoV-2 (PASC), post-acute COVID syndrome (PACS), vaccine, management, risk factor, drug repurposing

## Abstract

The pandemic of COVID-19 is the biggest public health crisis in 21^st^ Century. Besides the acute symptoms after infection, patients and society are also being challenged by the long-term health complications associated with COVID-19, commonly known as long COVID. While health professionals work hard to find proper treatments, large amount of knowledge has been accumulated in recent years. In order to deal with long COVID efficiently, it is important for people to keep up with current progresses and take proactive actions on long COVID. For this purpose, this review will first introduce the general background of long COVID, and then discuss its risk factors, diagnostic indicators and management strategies. This review will serve as a useful resource for people to understand and prepare for long COVID that will be with us in the foreseeable future.

## Introduction

Coronavirus disease 2019 (COVID-19) has escalated into an unprecedented global pandemic since the first case was identified in December 2019 1-3. By June 20^th^ 2022, more than 535 million individuals were infected with over 6.3 million deaths worldwide according to World Health Organization (WHO) 4. Apart from the severe morbidity and mortality in the first few weeks after infection, up to 70% COVID-19 survivors may experience long-term medical complications 5-7. The lingering symptoms after COVID-19 infection can last weeks to months, severely reducing the quality of life long after patients become virus-free. Such symptoms are generally reported as “long COVID” in common literature.

With so many affected patients, long COVID posts a global health challenge for the society 8. In the US, by fall 2021, cumulative loss from direct economic losses in conjunction with mortality, morbidity and relative metal health complications of COVID-19 is estimated at $16 trillion 9. The substantial burden of the pandemic should not be neglected. In just two years, a large volume of evidence has been accumulated on the pathological manifestation, treatments and preventative measures of long COVID 1. It is necessary for health professionals and the general public to gain more knowledge about the disease. Therefore, this review will present our current understanding of long COVID and highlight the potential management strategies and therapeutics.

## Acute COVID-19 and post-acute COVID-19 (long COVID)

**Figure 1:**
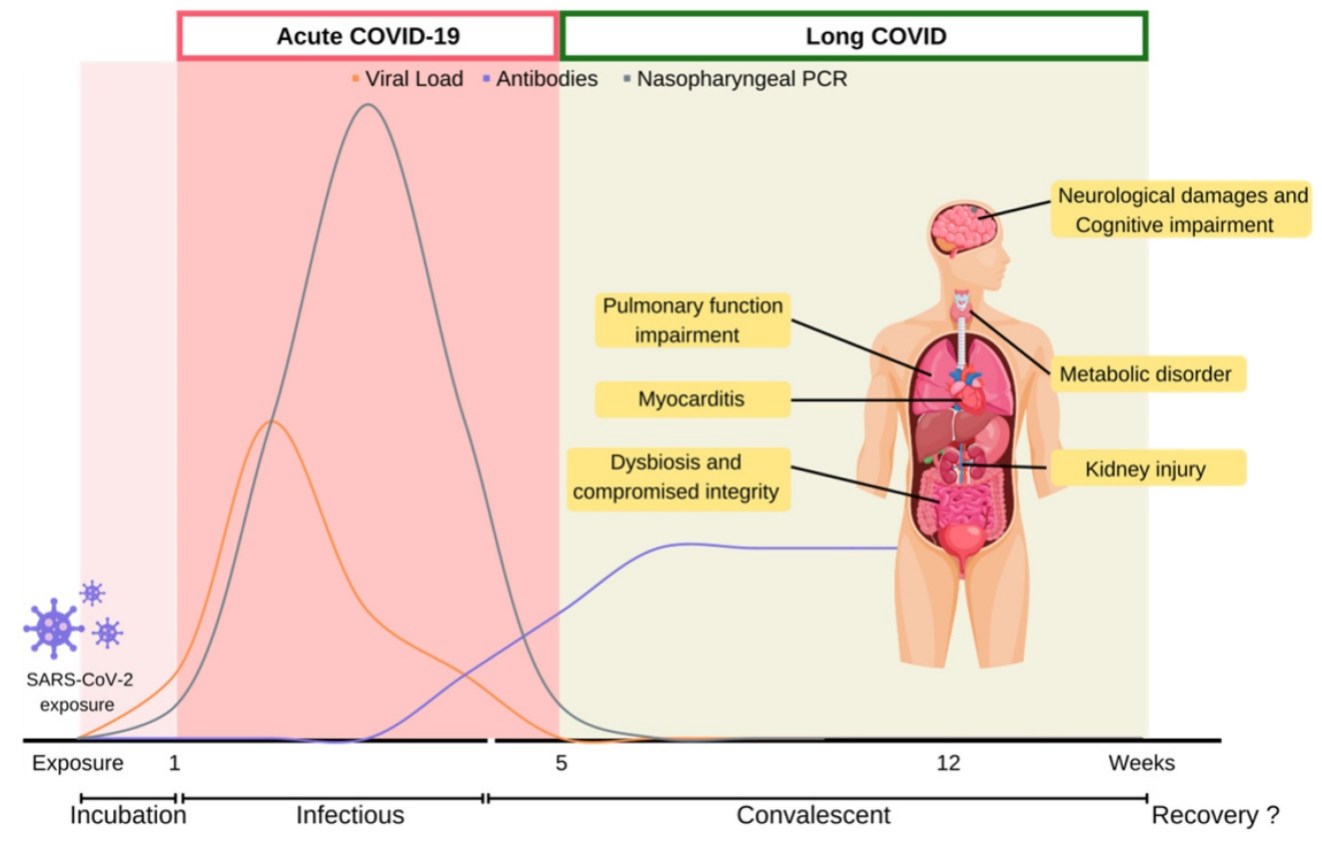
** Disease course of COVID-19.** COVID-19 disease course is depicted into multiple stages depending on viral counts and symptoms presented over time. Infected host undergoes acute-COVID-19 which lasts five weeks from symptoms onset, until competent virions become undetectable. Persistent symptoms and long-term complications beyond five weeks from COVID-19 infection is defined as long COVID. COVID-19: Coronavirus Disease 2019; PCR: polymerase chain reaction; SARS-CoV-2: severe acute respiratory syndrome coronavirus 2.

COVID-19 is caused by the infection of Severe Acute Respiratory Syndrome Coronavirus 2 (SARS-CoV-2), a singled-stranded RNA coronavirus 10. After the initial infection, patient would experience distinct symptoms in acute and post-acute stages over the course of the disease (Figure 1).

Acute COVID-19 stage generally refers to the initial five weeks after SARS-CoV-2 infect patients. When SARS-CoV-2 first enters the host respiratory tract, it initiates infection through the binding of the spike protein with Angiotensin-converting enzyme II (ACE2) receptor, and then utilizes transmembrane protease serine 2 (TMPRSS2) to enter host cells. SARS-CoV-2 hijacks the host cellular machinery for viral RNA replication and protein production. SARS-CoV-2 load usually reaches its peak upon symptom onset within the initial weeks of infection, and first becomes detectable by reverse transcription polymerase chain reaction (RT-PCR) within the first week of infection. At the peak of infection, a patient could carry 10^9^ to 10^10^ virions 11.

While the virion copy number is at its peak, the onset of infectious symptoms follows, which is referred to as acute COVID. Clinical manifestation of COVID-19 can range from asymptomatic, to severe fulminant illnesses leading to mortality. According to meta-analysis studies, mild flu-like symptoms such as fever and cough are commonly reported. In moderate to severe cases, onset acute respiratory distress syndrome (ARDS) may deteriorate rapidly and lead to the development of pneumonia and dyspnea. Severe pneumonia with ARDS progresses to pathological deteriorations including septic shock and multi-system organ failure, which account for high morbidity and mortality among severe infected patients 2, 5, 12-14. Acute COVID stage usually lasts five weeks till the viral load gradually declines from its peak to undetectable. During this period, viral infection often disables host cellular machineries, leading to functional impairments, even cell death. Subsequent cytokines recruitment often contributes to systematic hyperinflammation and leads to progression of thrombosis even multi-organ failure and death 2, 13. Majority of the confirmed cases (81-45%) were reported to be asymptomatic or mild disease at time of diagnosis. Approximately 25% of the initially asymptomatic individual would develop symptoms throughout COVID-19 acute stage 15. The Chinese Center for Disease Control and Prevention reported 14% of the COVID-19 patients were hospitalized with severe illness including dyspnea and 5% were reported as critical cases with disease progression to respiratory failure, shock, or multi-organ impairment 16. Additionally, critical or fatal risk of SARS-CoV-2 varied by age, pre-existing comorbidities and vaccination status. Pathophysiology of COVID-19 and its relevant biological disease course have been reviewed comprehensively elsewhere 12, 17.

About three weeks after initial detection by RT-PCR, the viral load becomes undetectable. This marks the end of acute COVID, but also the start of post-acute stage. COVID-relevant health issues could linger around as long COVID for years even in patients who are asymptomatic during the acute stage. Survivors of virus infections are at risk of developing physiological complications and multi-organ impairment. The subacute and sequelae effects of SARS-CoV-2 infection are called various names, such as “long COVID”, “post-acute COVID syndrome”, “post-COVID-19 condition” and “long-haul COVID-19”. In response to the absence of a consensus definition for long COVID, WHO has made a clinical case definition to the public in delineating long COVID. “Post-COVID-19 condition occurs in individuals with a history of probable or confirmed SARS-CoV-2 infection, usually 3 months from the onset of COVID-19 with symptoms that last for at least 2 months and cannot be explained by an alternative diagnosis.” 18 The National Institute for Health and Care Excellence (NICE) defined post-COVID-19 syndrome as “signs and symptoms that develop during or after an infection consistent with COVID‑19, continue for more than 12 weeks and are not explained by an alternative diagnosis” 19. Additionally, symptoms appearing during a timeframe of four-to-twelve-week post COVID-19 onset are regarded as ongoing symptomatic COVID-19. While the terminology for long COVID evolves as clinical evidence accumulates, the term “long COVID” will be used to discuss health issues during post-acute stage of COVID-19 in this review.

## Symptoms of long COVID

It is estimated that 31%-69% of COVID-19 survivors will experience long COVID symptoms after initial recovery from SARS-CoV-2 infection 20, 21. Generally, initial symptoms of long COVID symptoms include fatigue (29%), muscle pain, palpitations, cognitive impairment (28%), dyspnea (21%), anxiety (27%), chest pain, and arthralgia (18%) 22. Among the UK population, fatigue is most prevalent among long COVID patients (51%), followed by dyspnea (35%), arthralgia (25%) and concentration difficulties (25%). Correspondingly, a recent meta-analysis of 36 studies identified fatigue, cognitive impairment, joint pain, anxiety, and depression as primary clinical symptoms of long COVID 5. A massive international survey found fatigue, malaise and cognitive impairment as the most prevalence symptoms experienced among individuals with reported long COVID 23. Furthermore, cognitive related symptoms were reported to develop at later long COVID stages. While many cases were initially reported in hospitalized COVID-19 patients who experienced severe symptoms during the acute stage, long COVID symptoms have also been documented in non-hospitalized or asymptomatic individuals 24. Approximately 30% of non-hospitalized COVID-19 patients reported lingering symptoms 2 months after initial infections 25. Similarly, less than 1% of COVID survivors achieved complete recovery at 80 days after infection 26. The lingering symptoms of long COVID reflect chronical damages of multi-systemic organs. Such health conditions post a significant burden on the quality of life among COVID survivors (Figure 1) 27-29.

As a respiratory virus, SARS-CoV-2 infection leads to respiratory system dysfunction in long COVID. SARS-CoV-2 initially infects the alveolar epithelium, and induces chronic inflammation responses that trigger sustained production of inflammatory cytokines and reactive oxygen species (ROS) 17. Additionally, disruption of cellular integrity activates fibroblasts to deposit collagen and fibronectin, leading to fibrotic changes to lung tissue. In the long-term, viral-induced complement activation and subsequent disruption of coagulant pathways favor the development of prolonged inflammation and hypercoagulable state, predisposing the patient to the risk of thrombosis 30. National data from UK suggested that 36% of long COVID individuals experienced shortness of breath to a certain extent. 26% of long COVID individuals develop signs and symptoms of lung impairment 31. Respiratory abnormalities involving changes in total lung capacity and airway function have also been reported following COVID-19 infection 32. In addition, dyspnea is prevalent in COVID-19 survivors along with other respiratory symptoms, including chronic chough and reduction in exercise capacities 33.

Many long COVID patients also experience cardiovascular complications. The abundance of ACE2 receptors on cardiomyocytes provides a direct route for SARS-CoV-2 infection. Chronic inflammation of the cardiomyocytes can lead to myositis and cell death. Prolonged inflammation and cellular damage prompt fibrotic changes and reduce cell adhesion, all of which could lead to arrhythmia and the development of coagulopathy. In addition, inflammation of the autonomic nervous system might progress to postural orthostatic tachycardia syndrome (POTS) 34. Persistent myocardial inflammation and increased cardiac troponin level are commonly present in COVID-19 patients 2 months after diagnosis. Evidence have suggested increased risk of cardiovascular compilations following SARS-CoV-2 infection, even among non-hospitalized patients. In particular, cardiovascular risk was associated with the severity of infection during acute COVID-19 35. Correspondingly, a prospective observational study found that 32% COVID-19 survivors have cardiac damages 3 months after the onset of infection 36. 89% of long COVID patients reported cardiac associated symptoms, where 53% reported chest pain, 68% palpitations and 31% new onset of POTS 37.

Long COVID can also affect the central nervous system (CNS). Chronic neuro-inflammation activates glial cells which lead to neurodegenerative disorders. SARS-CoV-2 can permeate through the blood-brain barrier (BBB), and subsequent damage of the BBB permeability will further drive neuro-inflammation in the brain parenchyma. Pathological hyperinflammatory and hypercoagulable conditions may increase the risk of thrombotic events 38. Furthermore, hyper-inflammation in the brainstem may lead to autonomic dysfunction. CNS dysfunction in the brain may contribute to long-term cognitive impairment. Multiple studies have proposed that CNS disturbance in response to neuro-inflammation could be responsible for the neuropsychiatric abnormalities, including chronic malaise, fatigue, sleeping disorder, ageusia and anosmia (loss of taste and smell), PTSD, conative impairment, and even stroke 7. In a UK retrospective cohort study on 23,6379 confirmed COVID-19 cases, one in three subjects reported neuropsychiatric symptoms 6 months after SARS-CoV-2 infection 39. Specifically, subjects with severe acute COVID-19 were at higher risk for neuropsychiatric morbidity. Imaging studies using structural magnetic resonance imaging (MRI) and positron emission tomography (PET) imaging showed brain alterations among patients with cognitive impairment during subacute COVID-19 stage when compared to healthy controls 40-42. Combination of population and clinical data strongly suggest neurological involvement in long COVID.

SARS-CoV-2 induces both direct and indirect pathology which contribute to multi-organ dysfunction. Hyperinflammation of kidney tissues may activate the complement system, contributing to focal segmental glomerulosclerosis and glomerular involution 30. Acute kidney injury has been reported among discharged COVID-19 patients, and 35% of the recovered patients have reduced kidney function 43. SARS-CoV-2 triggers pancreatitis during acute-COVID. Pancreatic damage as observed in long COVID may be consequences from a combination of direct viral attack and indirect effect of systemic inflammation. The major SARS-CoV-2 receptor ACE2 is an endocrine regulator in the renin-angiotensin-aldosterone system, while the other SARS-CoV-2 receptor TMPRSS2 is found in pancreatic β cells. The viral infection directly impairs ACE2 and TMPRSS2-expressing cells, causing dysfunctions in renin-angiotensin-aldosterone system and disrupts metabolic homeostasis in the long term. Furthermore, systemic inflammation and corticosteroids administration increase the risk of bone demineralization and dermatologic complications 44.

Altered gastrointestinal system functions were reported in both acute- and subacute-COVID 22, 45. Expression of ACE2 receptor made the esophagus and enterocytes prone to direct viral damage 46. Upon viral clearance, prior hyperinflammatory state and microbiome imbalance may damage gastrointestinal integrity and function 47. Current findings have emphasized changes in the gut microbiota in patients following COVID-19 infection 48-50.

Considering the diverse manifestations of the disease, the full clinical spectrum of symptoms experienced by these patients over time needs to be catalogued to develop a better understanding of their underlying pathology. This would also further aid in the characterization of the various phenotypes of long COVID and the risk factors associated with its development, which will allow effective design of clinical trials with improved treatment options.

## Treatments and management of long COVID patients

Medical experts are using their best efforts to manage patients with long COVID. Although several guidelines on long COVID management have been released, there remains a large practical gap and specific treatments are not reviewed. While empirical evidence still awaits to inform clinical practice, a comprehensive review of clinical strategies adopted by clinicians will be valuable in guiding appropriate patient rehabilitation 51. It is important to optimize clinical outcomes by considering patient safety and enhancing sophisticated diagnosis and holistic assessment. Furthermore, novel therapeutics for treating organ specific dysfunction and long COVID related symptoms will guide treatment development. WHO have empathized research priorities in refining clinical characterization and developing therapeutics for long COVID. At the same time, medical professionals are exploring clinical approaches in identifying and managing long COVID.

Long COVID symptoms are presented heterogeneously, so patients need to be closely monitored. In order to develop effective treatment strategies, holistic assessment is necessary to consider pre-existing conditions and to identify specific symptoms. NICE outlines a set of evidence-based assessment and management approaches for treating patients with long COVID 52. The NICE guideline recommends clinical investigation of long COVID as early as 4 weeks after initial acute symptoms. In addition, the National Institute of Health and Care Research (NIHR) also issued recommendations on the evaluation of long COVID symptoms and prioritizing care for certain populations 53.

In general, current clinical practice adopted a symptom-based approach in managing long COVID. Comprehensive assessment through medical history and examination is essential. It is recommended to obtain a complete assessment including full blood count, renal function test, C-reactive protein, liver function test, thyroid function, hemoglobin A1c (HbA1c), vitamin D, magnesium, B12, folate and ferritin levels 52. The International Consensus Conference in Critical Care recommends adopting screening tests for prediction and identification of physical and mental impairment. A robust clinical care also requires additional assessments for appropriate referrals to specialists. Importantly, while long COVID is diagnosed, other non-COVID-19 related diagnosis should also be considered unless they could be excluded. Appropriate treatments are provided according to clinical symptoms 51. For patients presenting with cardiopulmonary symptoms, chest imaging, electrocardiography and pulmonary function tests should be considered. Oxygen supplementation is commonly provided for patients with dyspnea and during pulmonary rehabilitation. In particular, corticosteroid treatments have been showed to resolve pneumonia and improve clinical functions. Sufficient patient support and rapport building are essential for disease recovery 51.

### Mast cell activation syndrome (MCAS) and anti-histamine treatment

Previous findings have suggested the prevalence of mast cells activation syndrome in long COVID patients 54. It is suggested that immune disturbance from SARS-CoV-2 infection may lead to aberrant mast cell activation and further initiate cascades of inflammatory responses contributing to allergic flare-up 54, 55. Histamine antagonists have been used to relieve long COVID associated symptoms 55, 56. However, the interaction of antihistamines with viral-altered ACE2 pathways remains to be elucidated. One concern is that antihistamines can be easily assessed over the counter and abusive usage may lead to problems. Misuse of antihistamines will cause abnormal levels of circulating dopamine and may be associated with dementia in the long-term. Future studies and clinical trials are required to investigate antihistamines as therapeutic treatment for long COVID.

### Dietary supplements

In long COVID, chronic inflammation provokes multi-organ damage and exacerbates pre-existing conditions. Dietary supplements, such as vitamins and minerals, contain anti-inflammatory and anti-oxidative components, so they have become potential treatments for long COVID. A pilot study demonstrates that multivitamin supplements improve clinical symptoms among long COVID patients 57. In addition, a commercial plant extract supplement from *Panax ginseng* and *Eleutherococcus senticosus* effectively relieved post-COVID fatigue and improved health status in 201 long COVID patients 58. Nicotinamide ribose, a form of vitamin B3, is being examined for its effects of ameliorating cognitive dysfunctions and chronic fatigue in two clinical trials (NCT04809974, NCT04604704). Essential fatty acids, such as omega-3 (Eicosatetraenoic acid - EPA + docosahexaenoic acid - DHA), are also being examined for their functions in long COVID symptoms (NCT05121766).

Long COVID patients often have dysregulated lipid oxidation and lactate accumulation during physically active state, indicating compromised mitochondrial function 59. The mitochondrial dysfunction in long COVID shares similar symptoms as the ones observed in Myalgic encephalomyelitis/chronic fatigue syndrome (ME/CFS). Supplementation with Coenzyme Q10 (CoQ10) is found to reduce fatigue frequency and relieve oxidative stress among ME/CFS patients 60. Currently, high-dose CoQ10 treatment is being investigated in a Phase II clinical trial in long COVID patients (NCT04960215).

Dietary supplements may also have beneficial effect in modulating systemic inflammation and immunity. Natural flavonoids such as luteolin and quercetin are promising immunomodulatory agents which have showed inhibitory effects on mast cells 61. The influence of microbiota on immunity is well known, and long COVID leads to significant changes in gut flora 5, 49. Dietary pro-biotics and pre-biotics are being evaluated on their impacts on clinical symptoms, immune function and biomarkers in long COVID patients (NCT04813718).

### Other potential therapeutics

Because long COVID leads to systemic dysfunctions, various therapeutic strategies have been explored. Viral infection often compromises the immune system, which may increase the risk for opportunistic infections. Antibiotic and anti-viral compounds such as Azithromycin, Remdesivir and Favipiravir are being explored for their effectiveness in controlling long COVID (NCT04699097, NCT04978259, NCT04448119). In order to combat the extensive inflammatory state from COVID-19 infection, antibody treatments have also been studied, including Infliximab, Tocilizumab, Siltuximab, Anakinra and Leronlimab (NCT05220280, NCT04330638, NCT04330638, NCT04330638, NCT04678830). Meanwhile, antidepressants have been proposed to lessen long COVID symptoms by reducing peripheral inflammatory markers, thereby restoring immune function. Selective serotonin reuptake inhibitor and serotonin receptor modulator Vortioxetine is being examined in a clinical trial to treat long COVID (NCT05047952).

## Clinical indicators for long COVID

In order to effectively treat long COVID, it is crucial to have accurate diagnosis. Early diagnosis is essential for effective management and improved prognosis. Several clinical indicators have been developed to guide the clinical diagnosis of long COVID.

### Serum biochemistry

Acute COVID-19 infection disturbs the immune system response and predisposes a low-grade inflammation state which may continue over subacute phase. Systemic inflammatory markers were proposed as biomarkers for long COVID. For instance, D-dimer, c-reactive protein (CRP), interleukin-6 (IL-6), procalcitonin and neutrophil count found to be associated with persistence symptoms of long-COVID 33, 62-66. Cardiac, hepatic and renal abnormalities are also observed among patients with abnormal CRP, procalcitonin and neutrophil count levels 67. Analysis of endothelial function in 30 patients with long COVID reported distance ET-1 and RHI profile in long COVID patients 68. In addition, neurodegenerative indicators including amyloid beta, neurofilament light, neurogranin, total tau, and p-T181-tau were elevated among long COVID patients when compared to healthy controls 38.

The heterogenic nature of long COVID may justify the inconsistence of evidence in some studies 69-71. Furthermore, symptoms relapse and viral-shredding add challenges in diagnosing long COVID 72.

To this end, it would be very helpful for long COVID diagnosis if a set of biostable biomarkers are available independent of symptoms. A recent study found that the immunoglobin profile of IgM and IgG3 is linked with increasing risk for developing long COVID. In fact, IgM and IgG3 are secreted by B cells in response to interferon induction and IL-4 signaling. Impairment in interferon synthesis characterized by increased interleukin signaling may contribute to inefficient IgG isotype conversion, debilitating immunity regulation 73. As the pandemic evolves, research emerges toward a predictive model for post-acute sequelae of COVID-19 74.

### Gut microbiota

Immunomodulatory functions of gut microbiome are well documented in various disease pathogenesis 75. SARS-CoV-2 RNA can be detected from fecal samples during acute stage, indicating gastrointestinal (GI) involvement in COVID-19 pathology 46, 76. Recent studies reveal that gut microbiome diversity and composition are changed post COVID-19 infection 48-50. Increase in viral-induced cytokines may compromise intestinal integrity, facilitating the entry of bacteria and metabolites into circulation. Such dysbiosis triggers the innate immune responses, causing pulmonary dysfunction and secondary infections 77. Furthermore, integrative multi-omics profiling reveals that subjects with GI complications exhibit unique T cell signature during COVID-19 recovery 20. Current findings open opportunities for gut microbiota to be used as a therapeutical approach by priming inflammation responses. A prospective study of 106 subjects identified specific patterns of intestinal microbiome profile predicted long COVID symptoms. For examples, elevated levels of *Ruminococcus gnavus*,* Bacteroides vulgatus* and reduced levels of *Faecalibacterium prausnitzi* and butyrate-producing bacteria correlated with long COVID 49. Such intricate association may serve as a detection tool for the occurrence of long COVID.

## Proactive Management of long COVID

Considering that more and more people will likely be infected by emerging SARS-CoV-2 variants, long COVID will continue to affect the society immensely in the foreseeable future. It is critical for people to take proactive actions to relieve its potential impacts. In doing so, we need to understand the risk factors of long COVID, control the risk factors and avoid questionable practices.

### Risk factors of long COVID

Ever since long COVID was first reported, much attention has been paid to identifying its risk factors. The understanding of risk factors allows people to correlate long COVID with various determinants such as pre-existing conditions, age, medical treatments, genetics and lifestyle. The specific correlations will guide clinical diagnosis and management of long COVID. Symptoms in long COVID arise from multisystem damage, a holistic approach should be considered when identifying at-risk individuals at early phase of the disease 53.

#### 1. The severity of Acute COVID infection

**Figure 2:**
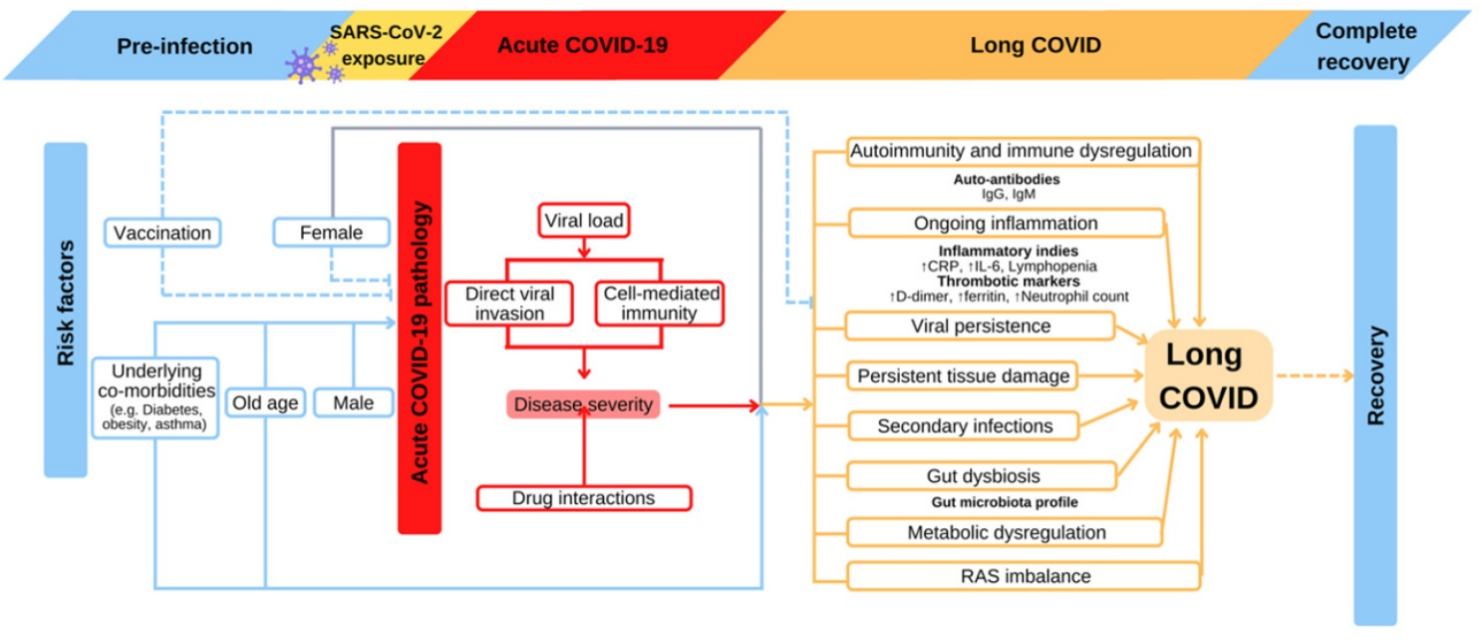
** Proposed risk factors and relevant pathophysiological consequences of long COVID development.** Old age, pre-existing comorbidities, and incomplete COVID-19 vaccination are well-established risk factors of acute-COVID-19 severity, which subsequently increase the risk for long COVID. Altered clinical parameters such as immunoglobins, inflammation cytokines, and microbiome profile could reflect the progression of long COVID. Such predisposition may also have an impact on recovery. COVID-19: Coronavirus Disease 2019; CRP: c-reactive protein; IgG: immunoglobulin G; IgM: immunoglobulin M; IL-6: interleukin 6; RAS: renin-angiotensin system; SARS-CoV-2: severe acute respiratory syndrome coronavirus 2.

Long COVID was first reported in hospitalized patients requiring mechanical ventilation during acute stage, suggesting the symptoms at this stage correlates with the prevalence and severity of long COVID 69, 78, 79 (Figure 2). It is reported that viral load during acute COVID correlates with the severity of long COVID manifestation 20. Early viral clearance seems to be protective from long COVID responses. Low viral load or early viral clearance probably leads to lower grade inflammatory reactions during acute phase, which results in less damage to the host in long COVID. The COVID Symptom Study shows that symptom quantity during the initial infectious week is predictive of long COVID duration 74. Multiple population-level survey studies also demonstrate the positive correlation between acute disease symptoms with long COVID 24, 80. Among hospitalized population, persistent symptoms are more frequently reported in patients with history of intensive care unit (ICU) admission or mechanical ventilation 81, 82.

In the past three years, the continuous viral evolution leads to the emergence of numerous SARS-CoV-2 variants in global populations, which challenges the effectiveness of public health in diagnosis, vaccination, and treatments. Five variants of concern (VOC) have been widely reported, and they are Alpha, Beta, Gamma, Delta and Omicron variants 83. Because of their specific mutations in viral genome, different variants have various transmissibility and virulence, which affects both Acute COVID-19 and long COVID in patients. Currently, Omicron variant is the dominant variant with enhanced transmissibility since February 2022, replacing Delta variant that was most prominent in 2021. A recent study showed that Omicron variant caused lower risk in developing long COVID in comparison to Delta variant among infected patients 84. The finding is probably associated with the fact that Omicron variant generally caused less severe symptoms than Delta variant at Acute stage. However, because of its high transmissibility, more people will likely be infected by the Omicron variant. In this sense, Omicron variant associated long COVID will remain a major challenge for people in foreseeable future.

#### 2. Pre-existing conditions

Pre-existing conditions correlate with the severity at acute stage, and are risk factors for developing long COVID. In a recent study, a combined analysis of UK primary care electronic health records and 10 population-based longitudinal studies revealed multiple risk factors for long COVID, including age, female gender, poor general health, asthma, and being overweight or obese 85. In a prospective cohort study of 215 participants, long COVID developed in 94% of participants with a history of asthma bronchial, compared to 59% in those without 62. The complex interactions of asthma severity, underlying conditions, as well as corticosteroid administration should be considered when managing long COVID. In addition to asthma, other pre-existing conditions were also reported to be long COVID risk factors. Pre-existing type 2 diabetes increase the risk of long COVID, as chronic inflammation associated with insulin resistance leads to a more profound immune responses during acute SARS-CoV-2 infection and subsequent COVID sequala 20. Poor mental health is associated with 50% increased risk of developing long COVID and an increase in its relative severity 85, 86. Interestingly, history of Epstein-Barr virus viremia might increase the risk for long COVID as suggested from single-cell sequencing of long COVID subjects 20. With ongoing research, additional medical conditions may be identified as risk factors for long COVID.

#### 3. Age

Advanced age is a main risk factor for the severity of acute infection, as well as the risk of long COVID. The COVID Symptom Study identified advanced age as a risk factor for long COVID 7. Approximately one in five COVID survivor of over 70 years of age reported having lasting symptoms 87. In contrast, self-reporting data from the UK's Office for National Statistic suggested subjects aged from 35 to 69 have the highest prevalence in experiencing long COVID 31. Discrepancy of such results warrants further research to elucidate underlying mechanisms and identify long COVID patterns in different age groups. Considering that older patients are more likely to have pre-existing conditions and develop more severe acute responses, the increased risk of long COVID with advanced age may be a secondary effect.

#### 4. Biological sex and sex hormones

Clinical data from epidemiological studies have drawn the attention toward the sex-discrepancy in long COVID 88. In general, females under age 50 are five times more likely to develop long COVID symptoms post-discharge when comparing to male COVID-19 patients 89. Multiple studies have observed higher prevalence of long COVID in female subjects than male 43, 81, 86, 90, 91.

Ovarian abnormality following HIV and Hepatitis B/C infections has been previously reported 92. The abundance of ACE2 in ovarian granulosa cells and the hormonal disruption from SARS-CoV-2 may both contribute to ovarian dysfunction 25. Specifically, perimenopausal and menopausal women are more prone to long COVID. Virus-mediated ovarian hormone disturbance interferes with systematic homeostasis reflects the inflammatory disorder over disease course in anticipating long COVID. However, overlapping symptoms of long COVID and menopause-related conditions pose additional challenges in the diagnosis and management of long COVID in this population.

Paradoxically, male sex is a risk factor for acute infection, but fewer long COVID cases are reported in male patients. It is noticed that long COVID studies are largely derived from self-reported data. Selection and reporting biases may arise from such methodological limitations. It is possible that part of the sex-associated long COVID prevalence may be linked to sex-dependent self-reporting. Meanwhile, sex-dependent discrepancy in medical care already exist in pre-COVID-19 era, so more complicated social issues could also lead to the increased number of female long COVID patients.

## Proactive measures to relieve the potential impacts by Long COVID

**Figure 3:**
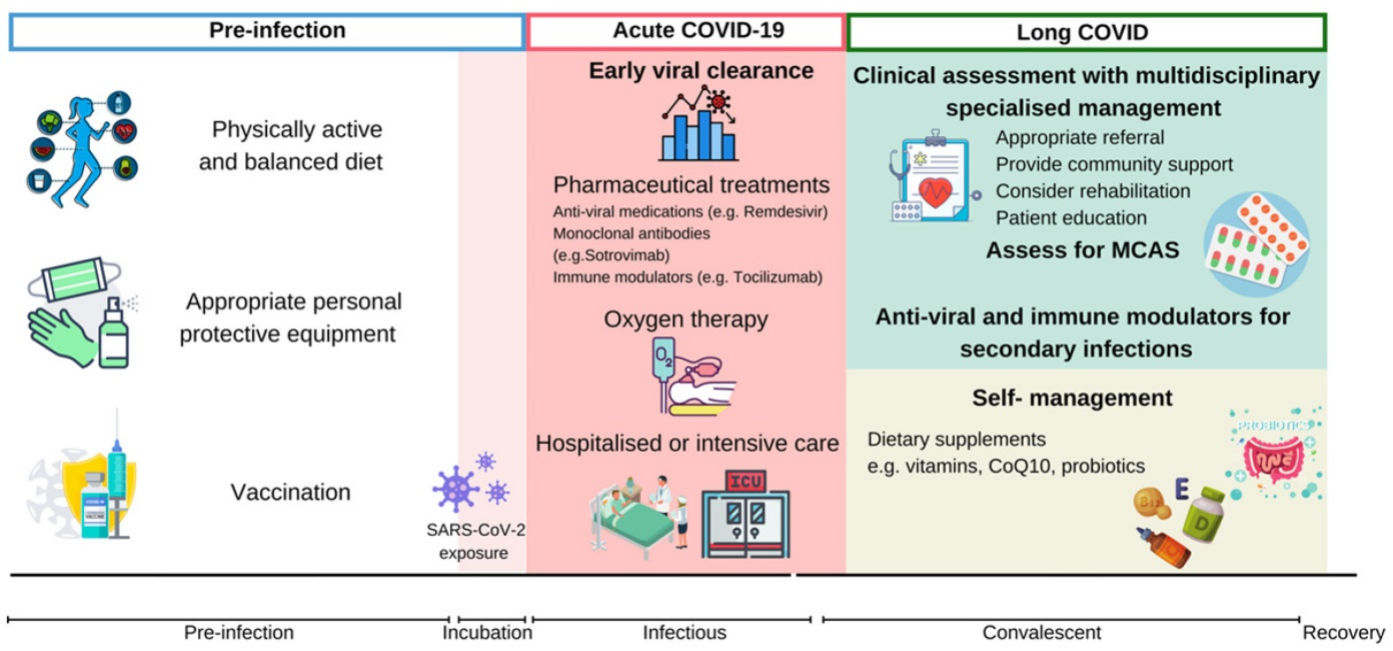
** Proactive measures and treatment strategies invovled in relieving long COVID.** Management of long COVID should be prioritized throughout the course of disease. Sufficient nutrients from a well-balanced diet, physical activity, appropriate PPE and COVID-19 vaccination are protective measures against SARS-CoV-2 infection and the progression of long COVID. Early viral clearance by preproperate clinical practice during acute-COVID-19 may alleviate viral infection and prevent poor clinical outcomes. Integrated management plan should be provided to individuals with long COVID, depending on assessments and targeted management. CoQ10: coenzyme Q10; COVID-19: Coronavirus Disease 2019; CRP: c-reactive protein; MCAS: mast cell activation syndrome; SARS-CoV-2: severe acute respiratory syndrome coronavirus 2.

Considering the enormous impact of SARS-CoV-2, the health burden of long COVID should not be neglected. The general public should consider proactive approaches to prevent long COVID. Summarized proactive measures are described in Figure 3.

### 1. Vaccination

Considering the impact of long covid to individual's wellbeing and society, it is important to take preventive measures besides trying to avoid infection. In the past two years, various vaccines have been developed for SARS-CoV-2. Although vaccines do not prevent infection, they significantly suppress morbidity and fatality. Two recent studies compared the long covid symptoms between unvaccinated patients and vaccinated patients in Israel 93 and the UK 94. Both demonstrated that vaccination is strongly associated with the decrease of long COVID related symptoms. In fact, one of the studies showed that vaccinated people are no more likely to report long COVID symptoms than individuals without prior viral infection. In Switzerland, about 40% of death in connection with SARS-CoV-2 infection had not received COVID-19 vaccination in contrast to ~ 10% of death shared by individuals who had received at least single dose of vaccination 95. These data suggest that vaccination is beneficial in controlling both acute and long COVID-19 symptoms.

The fifth wave of COVID-19 outbreak in Hong Kong has alarmed the COVID-19 vaccine hesitancy and associated factors in the community 96, 97. Vaccination coverage in Hong Kong declined with age, with 49% of individuals aged above 60 reported receiving at least two doses of COVID-19 vaccine 98. The COVID-19-associated mortality was prevalent among unvaccinated individuals aged above 60 years. The increased risk of COVID-19 severity among older population groups suggested relative risk for long COVID. Data from Hong Kong highlighted the important to implement strategies to boost vaccine coverage, especially among older adults.

In addition to vaccines specific to SARS-CoV-2, people reported that other unrelated vaccine might also provide protective effects. A study on health care workers showed that recent influenza vaccination reduced the risk of SARS-CoV-2 infection and COVID-19 severity 99. A retrospective study also showed that influenza vaccination significantly decreased the risks of sepsis, stroke and deep vein thrombosis associated with long COVID, and reduced the admission to Emergency Department and Intensive Care Unit weeks after initial infection 100. Besides from the novel COVID vaccination, the bacillus Calmette-Guérin (BCG) vaccine against tuberculosis holds general protection from wide range of infectious diseases 101, 102. In the era of COVID, the BCG vaccine has been proposed against SARS-CoV-2 severity where BCG vaccination is shown to have negatively associated with COVID-19 mortality 103, 104. One study showed that BCG vaccine against COVID-19 was significant in young population, but was not as effective in older population 105. However, another study from South Africa reported no protective effect by BCG vaccination against COVID-19 infection, severity and related mortality 106. Above contradictory reports may contain analytic complications such as confounding mortality with tuberculosis and differences in demographics and diagnosis, so more human and animal studies are necessary to reveal whether influenza and BCG vaccines could stimulate the basal immune defense against SARS-CoV-2^107^, ^108^. In the absence of solid evidence, influenza and BCG vaccines are not recommended as a preventative vaccination for COVID-19^109^.

### 2. Anti-inflammation treatment

Immunological aberrations owning to molecular mimicry induce autoantibody production which stimulates T cells and leads to tissue damage during acute COVID-19 infection 110. Therefore, combating inflammation responses is critical in managing viral manifestation as well as clinical sequelae of SARS-CoV2. Dexamethasone is commonly used to treat inflammation in acute COVID-19 patients. Dexamethasone-treated COVID-19 patients were less likely to experience long COVID symptoms at 8-month follow-up in an observational study 111. This study suggests that disease treatment approach for acute COVID could have profound impact on patients' wellbeing in the long run.

### 3. Nutritional control and lifestyle modifications

Nutritional management plays an important role in the management of chronic illness, and appropriate nutrition may mitigate the manifestations of viral infections 112-114. Moreover, disparities in SARS-CoV-2 incidences across population indicate the plausibility of nutrition-related epigenetic variations among different populations 115. For instance, high fat intake is found to downregulate the expression of ACE2 in animal models 116. In addition to dietary patterns, lifestyle interventions and sleeping habits also have primordial function in shaping innate immune responses to external stimuli 117.

A meta-analysis of 361,934 participants reported a significant association between vitamin D insufficiency and increased risk of SARS-CoV-2 infection (OR = 1.43, 95% CI 1.00-2.05) 49. This suggested the protective roles of vitamin D against SARS-CoV-2 118. Multiple observational studies and clinical trials have established the beneficial effect of vitamin D supplementation in preventing respiratory infections 119-121. For instance, numerous ongoing clinical trials are evaluating the clinical outcomes of vitamin D supplementation in the context of COVID. generally, vitamin D is derived primarily from UVB radiation exposure and dietary intake 122. Both UV-synthesized and ingested vitamin D then undergo two hydroxylation reactions to become activated as calcitriol. Activated vitamin D is involved in multiple immune functions, including the maintenance of barriers, antigen presentation, innate immunity and adaptive responses. Furthermore, the regulatory roles of vitamin D in the renin-angiotensin-aldosterone system (RAAS) may contribute to its therapeutic potential in mitigating SARS-CoV-2 pathogenesis. Despite the association between vitamin D deficiency and SARS-CoV-2 infection, evidence to support vitamin D supplementation for long COVID management is still lacking. Although recent study did not find an association between vitamin D levels and persistent long COVID symptoms, vitamin D deficiency is known to be associated with fatigue and muscle weakness. Additional research is warranted to explore the relationship between vitamin D and the pathology of long COVID. Optimal vitamin D intake from sunlight exposure and vitamin D rich food (fish, mushroom, and vitamin d-fortified foods) may have proactive effects in preventing COVID-19 infection and relative long COVID risk.

Probiotics are well known to reinforce immunity and counteract inflammation by restoring the gut microbiota. A growing body of research supports the beneficial role of probiotics in lung and mental health by modulating the gut-lung and gut-brain axes 45. Recently, *Lactobacillus plantarum* is found to exhibit antiviral effects in SARS-CoV-2 infected intestinal-epithelial cells 123. Although the beneficial role of probiotics is extensively reported, the interactions of probiotics supplementation for COVID-19 management are not fully elucidated 124, 125.

On the other hand, antibiotic administration has been reported to disrupt gut microflora which resulted in declined prognosis in mouse models of lung infection 47. Antibiotic use can have several negative effects on the gut microbiota, including reduced species diversity, altered metabolic activity, and the selection of antibiotic-resistant organism. The risks associated with the use of antibiotics for managing long COVID requires continued research.

### 4. Controversial practices

Repurposed therapeutics, such as ivermectin and hydroxychloroquine are widely available in many countries, which may interfere with proper treatments such as vaccine and dexamethasone. Such situation may lead to increased risk for the development of long COVID.

Anti-parasitic drug, Ivermectin, is a candidate drug during the COVID-19 pandemic. Given the availability and affordability of Ivermectin, abusive usage of ivermectin was reported in some nations as a control effort for COVID-19. However, evidence supporting the utilization of these drugs relied on laboratory data. Clinical data supporting the administration of ivermectin had significant methodical limitations 126. Meanwhile, Chloroquine/hydroxychloroquine, an anti-malarial treatment 127, has been wildly utilized and studied since the initial outbreak of COVID-19. Preliminary data reported enhanced viral clearance among COVID-19 infected patients with hydroxychloroquine compared with standard treatment 128. However, as research evolved, the use of hydroxychloroquine became controversial due to the lack of well-designed studies and potential bias from media propaganda 129. The adverse outcomes from the administration of such therapeutics in the context of long COVID have not been comprehensively reviewed. Furthermore, NIH recommended against the use of the aforementioned drugs for the treatment and prevention of COVID-19.

## Conclusion

SARS-CoV-2 has been evolving quickly in the past two years, and multiple variants have gained increased abilities to infect patients or evade the protection by vaccination. Human species will likely co-exist with the virus for many years to come, so long COVID will remain a global challenge to health care system and economy. In order to effectively deal with long COVID, it is important to raise public awareness of its risk factors and take proper management options. With increasing knowledge of COVID-19, the society will be able to protect people's interests if everyone acts together.
